# Simultaneous Methylation-Level Assessment of Hundreds of CpG Sites by Targeted Bisulfite PCR Sequencing (TBPseq)

**DOI:** 10.3389/fgene.2017.00097

**Published:** 2017-07-13

**Authors:** Kyuheum Jeon, Byungkuk Min, Jung S. Park, Yong-Kook Kang

**Affiliations:** ^1^Development and Differentiation Research Center Korea Research Institute of Bioscience & Biotechnology, Daejeon, South Korea; ^2^Department of Functional Genomics, Korea University of Science and Technology Daejeon, South Korea

**Keywords:** DNA methylation, cancer, LIFR, targeted NGS, sequencing, diagnosis

## Abstract

Methylated-DNA sequencing technologies are producing vast amounts of methylome data from cancer samples, from which cancer-associated differentially methylated CpG sites (cDMCs) are continuously identified and filed. The inclusion of as many cDMCs as possible helps improve the accuracy of cancer diagnosis and sometimes identify cancer subtypes. However, the lack of an established method for the analysis of 100s of cDMCs practically impedes their robust use in clinical medicine. Here, we tested the availability of targeted bisulfite-PCR-sequencing (TBPseq) technology for the assessment of methylation levels of a myriad of CpGs scattered over the genome. In randomly selected 46 cancer cell lines, multiplexed PCR yielded a variety of amplicons harboring 246 CpGs residing at promoters of 97 cancer-associated genes, all of which were sequenced in the same flow cell. Clustering analysis of the TBPseq-assessed methylation levels of target CpGs showed that the lung and liver cancer cell lines correlated relatively strongly with each other while they weakly correlated with colon cancer cells. CpGs at the *LIFR* gene promoter, which are known to be hypermethylated in colon cancers, indeed were heavily methylated in the tested colon cancer cells. Moreover, the *LIFR* promoter hypermethylation was found in colon cancer cells only, but not in biliary tract, liver, lung, and stomach cancers cell lines. A meta-analysis with public cancer methylome data verified the colon cancer specificity of *LIFR* promoter methylation. These results demonstrate that our TBPseq-based methylation assessment could be considered an effective, accurate, and competitive method to simultaneously examine a large number of target cDMCs and patient samples.

## Introduction

The potential role of epigenetic processes in human diseases is exemplified by aberrant DNA methylation in cancer (Heyn and Esteller, 2012). Hence, a key challenge in this field is an ability to detect these alterations genome-wide in high-resolution within a large number of samples to identify cancer associations. Several effective technologies have been widely used to characterize genome-scale patterns of DNA methylation: (1) whole-genome bisulfite sequencing (WGBS), which treats DNA with bisulfite and is followed by next-generation sequencing (NGS) technology, providing a complete overview of CpG methylation at base-pair resolution and an unbiased assessment of the profile of DNA methylomes (Lister et al., 2008, 2009; Laurent et al., 2010); (2) Infinium HumanMethylation 450 BeadChip, a DNA methylation array technology, which assays approximately 450,000 individual CpG sites that cover 99% of all RefSeq genes, allowing the high-resolution DNA methylation profiling of human samples (Bibikova et al., 2011; Sandoval et al., 2011); (3) reduced representation bisulfite sequencing (RRBS), which reduces the representation libraries by DNA digestion using a methylation-insensitive restriction enzyme such as *Msp*I, is suitable for obtaining information from most CpG islands (Meissner et al., 2005) and has been used to identify changes during cell differentiation (Bock et al., 2011); and (4) methylated DNA binding domain sequencing (MBD-seq) and methylation DNA immunoprecipitation sequencing (MeDIP-seq), which combine the advantages of NGS and enrichment of methylated DNA regions by immunoprecipitation (Down et al., 2008; Serre et al., 2010), are suitable for covering large parts of the genome in a quantitative manner and have been successfully used to identify aberrantly methylated disease-related CpGs (Feber et al., 2011).

These methylated-DNA sequencing technologies have been used to produce vast amounts of methylome data from 1000s of cancer samples worldwide, from which innumerable cancer-associated differentially methylated CpGs (cDMCs) have been identified. Although the cDMCs identified this way are continuously being filed and validated as cancer biomarkers (Model et al., 2007; Li et al., 2009, 2012; Hinoue et al., 2012; Khamas et al., 2012; Nikolaidis et al., 2012; Simmer et al., 2012; Naumov et al., 2013; Mitchell et al., 2014), the practical application of the cDMCs to clinical diagnosis has been rare, and the use of them in a pack of 10s or 100s in a single assay is even rarer. The inclusion of as many cDMCs as possible can help increase the diagnostic capability, and identify and classify cancer subtypes from an epigenetic perspective. Once a pack of cDMCs are set as cancer biomarkers through scrupulous validation processes, they are ready for use in probing into methylation states of cancer samples. Compared with the whole genome-scale sequencing and the analysis of the correspondingly large amount of data, this target-based approach would be much cost effective, less time-consuming, less labor-intensive, and thus far more competitive. Owing to all these benefits, the targeted methylation analysis method could undoubtedly be considered a better choice for cancer diagnosis. Unfortunately, however, there has been no established technology until date to analyze 10s or 100s of CpGs at a time, which serves as a major barrier to the practicality and high diagnostic capabilities of large-scale cDMC use in clinical medicine.

In this study, we tested the applicability of a targeted bisulfite PCR sequencing (TBPseq) technology, a promising approach for the methylation analysis of a large-size pack of CpGs. Our study was designed with an aim to show that some CpGs previously known as biomarkers for certain cancers could indeed be exposed as being differentially methylated in the same type of cancer cells among different cell lines examined. To confirm this possibility, we assessed the methylation frequencies of 100s of CpGs, which have been known to be cancer-related, in 46 cell lines representing five different cancer types (biliary tract, colon, liver, lung, and stomach cancers). We have succeeded in providing evidence that our TBPseq method is accurate and effective for the methylation analysis of a large number of samples and target CpGs at a time.

## Materials and Methods

### Ethics Statement and Cell Culture

This study was carried out in strict accordance with the recommendations in the Guide for the Care and Use of Laboratory Animals of the National Livestock Research Institute of Korea. The protocol was approved by the Committee on the Ethics of Animal Experiments of the Korea Research Institute of Bioscience and Biotechnology.

Colon cancer cells were cultured in RPMI-1640 media (Gibco) containing 10% heat-inactivated fetal bovine serum (RMBIO, United States), 0.5% non-essential amino acids, 100 U/ml penicillin, and 0.1 mg/ml streptomycin at 37°C and 5% CO_2_. CCD-18co cell was cultured in DMEM media (Gibco) containing 10% heat-inactivated fetal bovine serum (RMBIO, United States), 0.5% non-essential amino acids, 100 U/ml penicillin, and 0.1 mg/ml streptomycin at 37°C and 5% CO_2_.

### Reverse Transcription-Polymerase Chain Reaction (RT-PCR)

For reverse transcription-polymerase chain reaction (RT-PCR), total RNAs were obtained from cultured cells using RNeasy mini kit (Qiagen) and were used to synthesize cDNAs using reverse transcriptase (Superscript III, Invitrogen) as described elsewhere (Cho et al., 2015). PCR was performed with h-taq DNA polymerase (SolGent) in the following conditions: 15 min of enzyme activation at 95°C followed by 40 cycles of 95°C for 20 s, 55°C for 30 s, and 65°C for 1 min. The list of PCR primers is as follows: 5′-CAGGGGATGGCAAGATAG-3′ and 5′-TCTTTTATTGTCCACCATCC-3′ for *LIFR*; 5′-AACAGTGC CTTGAGGAGAG-3′ and 5′-GGGCTGTTTAGGTAATTCG-3′ for *LIFR-AS1*; 5′-AATCCCATCACCATCTTCCA-3′ and 5′-TG GACTCCACGACGTACTCA-3′ for *GAPDH*.

### Combined Bisulfite Restriction Analysis (COBRA)

Genomic DNAs extracted from culture cell lines were treated with bisulfite using EpiTect kit (Qiagen), as described elsewhere (Cho et al., 2014). The bisulfite converted gDNAs were used as templates in PCR to amplify the region of interest harboring either *Taq*I (5′-TCGA-3′) or *Bst*UI (5′-CGCG-3′) sequence. The list of PCR primers is as follows: 5′-TTTTTAGAAGGTTATGGAAG-3′ and 5′-CTC TCCAACTAATTTCATTT-3′ for *LIFR*; 5′-TTAAGTGAAGA AATTTTGAA-3′ and 5′-TTATCTCCAAATATCACAAA-3′ for *MLH1* (1); 5′-GTTTTTATTGGTTGGATATT-3′ and 5′-AA ATACCAATCAAATTTCTC-3′ for *MLH1* (2); 5′-AGTTA TTTTAGGGGAAGTAA-3′ and 5′-AAAACCCTACAATTA AACAC-3′ for *HOXA11*. The resulting PCR products were digested with either *Taq*I or *Bst*UI and resolved on 2% agarose gel.

### Bisulfite Treatment and Multiplex PCR

Genomic DNAs (gDNA) were extracted from cancer cell lines and were treated with bisulfite using EpiTect Bisulfite Kit (Qiagen) according to the manufacturer’s instruction. Multiplexed PCR was prepared with 113 pairs of primers in total. To design the multiplex PCR primers, promoter sequences between 1 kb upstream and 500 bp downstream from transcription start sites (TSSs) of multiple target genes were extracted using the ‘bedtools getfasta’ (Quinlan, 2002), and then the C→T and G→A converted target DNA sequences were generated from the extracted sequences by ‘bismark_genome_preparation’ (Krueger and Andrews, 2011). With these sequences as input templates, we ran a web-based ‘Batchprimer3’ engine (You et al., 2008) to obtain primers in batches which were then split into six subsets after experimental verifications of their proper operations by gel electrophoreses of multiplex PCR products from various combinations of primer sets. The list of primer pairs and their amplicon sequences is presented in the Supplementary File 1. Multiplex PCR was performed with each primer group using h-Taq DNA polymerase (SolGent) in the following conditions: 15 min of enzyme activation at 95°C followed by 50 cycles of 95°C for 20 s, 46°C for 1 min, and 65°C for 2 min (Oh et al., 2015; Park et al., 2017).

### Library Construction for Illumina Sequencing

Entire amplicons obtained from eight rounds of multiplex PCR were pooled together in equal volumes. For sequencing library construction, we performed a series of enzymatic reactions such as 5′-end phosphorylation, adaptor ligation, and additional cycles of PCR to attach barcode and other modules. 5′-end phosphorylation was performed with 1 μg of pooled amplicons using T4 polynucleotide kinase (NEB) at 37°C for 30 min. 5′-end phosphorylated amplicons then were ligated with 15 μM of home-made Illumina adaptors by incubating them at RT for 1 h. Finally, adaptor ligated amplicons were amplified using index and universal primers by DNA polymerase (SolGent) for indexing in the following conditions: 15 min of enzyme activation at 98°C followed by 25 cycles of 98°C for 10 s, 65°C for 30 s, and 72°C for 30 s (Min et al., 2016). Every intermittent purification was conducted using Expin^TM^ PCR SV purification kit (GeneAll). The equal amount of barcoded libraries were pooled, and they were applied to a parallel deep sequencing in a single flow cell using Illumina Hi-seq 2500.

### Detection of Cancer-Related Differentially Methylated CpG (cDMC) and Statistical Analysis

Raw read sequences were pre-processed to remove Illumina adapter sequences and low quality bases using “trim_galore,” and the trimmed reads were mapped on the C→T and G→A converted target sequences were generated using “bismark genome preparation.” Target CpG methylation levels were measured by “Bismark methylation extractor,” and CpGs with the low read coverage (<100) including amplification failed targets were filtered out. Following data analyses were conducted in R statistical environment^1^ unless stated otherwise. A dataset containing normalized DNA methylation levels of target CpGs was generated using “DESeq.” To compare overall methylation pattern among cancer types, principal component analysis (PCA) was conducted using “prcomp” function and the results were illustrated by “plotPCA” function. Pearson correlation coefficients (*r*) between individual cancer samples and cancer groups were calculated by “cor” function and a scatter matrix was produced using “heatscatter” function in “LSD” package. For the detection of cDMCs, relative methylation levels between cancer types were calculated, and CpGs showed statistically significant differences (FDR < 0.0001 and fold-change > 2) were determined by “DESeq.” For a meta-analysis, cancer type-specific DNA methylome data generated by Infinium 450k array (Illumina) for colon, lung, liver, and stomach cancer samples were downloaded from GDC^2^. Methylation levels (beta-values) of target CpGs were extracted from the methylome data and compared them between different cancer types. Statistical significance was calculated using Wilcoxon rank sum test. All plots were generated using R and MS Excel.

## Results

### Evaluation of the Targeted Bisulfite PCR Sequencing Method

**FIGURE 1:**
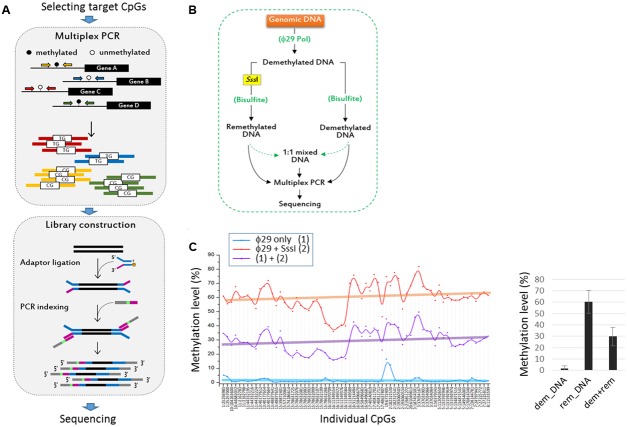
Evaluation of the targeted bisulfite PCR sequencing (TBPseq) method. **(A)** Illustration of TBPseq method. Colored horizontal arrows denote primers for target amplification. Amplicons have methylated (5′-CG-3′) or unmethylated (5′-TG-3′) sequences for target CpGs, the ratio of which is used for calculating methylation frequencies of the target CpGs. A total of 113 primer pairs were split into six groups of about 20 pairs for separate multiplexed PCR. **(B)** Strategy for evaluating the TBPseq method. The whole genomic DNA of 293T cells was amplified using ϕ29 DNA polymerase. A half of the resulting newly synthesized and unmodified (demethylated) DNA was re-methylated *in vitro* using CpG dinucleotide-specific *Sss*I methylase. The remethylated and demethylated DNA fractions were equally treated with bisulfite and used as template in multiplex PCR with 20–25 primer pairs per reaction. Multiplex PCR was additionally performed with an equal mixture (1:1 mixed DNA) of the remethylated and demethylated DNA templates. The three different groups of amplicons were modified and differentially barcoded for Illumina sequencing. **(C)** Methylation levels of target CpG sites. Each CpG site has three different methylation levels that were obtained from demethylated DNA (ϕ29 only, blue), remethylated DNA (ϕ29 + SssI, red), and an equal mixture of them (purple). In the right panel, the mean methylation levels of target CPGs in the three DNA groups are shown (error bars, standard deviation). Individual CpG sites are indicated by their genomic position as “chromosome (chr) number:coordinate” using GRCh37/hg19 as reference genome.

To assess the methylation levels of a large number of CpG sites *en bloc*, we designed a method for TBPseq, a multiplex PCR-combined targeted sequencing strategy, as illustrated in **Figure 1A**. From the literature and public cancer DNA methylation databases, we randomly selected 246 CpG sites at the promoters of 97 genes that are known to be frequently associated with various types of cancers. Cytosine-to-thymine converted versions of sequence stretches as bisulfite-treated template DNAs were fed to the BatchPrimer3 (You et al., 2008) to design primer pairs for multiplex PCR (Supplementary File 1). The resulting PCR products were processed for deep sequencing. Supplementary Figure S1 shows representative results of multiplex PCR and mapping of the sequenced amplicon reads harboring individual target CpGs.

We first evaluated whether TBPseq could output the amplicon data accurately and unbiasedly and was sufficient to reflect the methylation states of input DNA. As outlined in **Figure 1B**, whole genomic DNA of a 293T cell was repeatedly copied using the ϕ29 DNA polymerase. The resultant demethylated DNA was re-methylated using a CpG-specific *Sss*I methylase, treated with bisulfite, and then used in the multiplex PCR as a template. The results from amplicon sequencing showed that the mean methylation level was 60.4 ± 9.9% when the *Sss*I-remethylated DNA template was used, whereas the levels were 1.6 ± 2.5% and 29.8 ± 8.2% when the demethylated DNA template only or an equal mixture of remethylated and demethylated DNA, respectively, was used (**Figure 1C**). The 60% methylation level obtained from the use of the remethylated DNA indicated an incomplete methylation, possibly by insufficient *Sss*I enzyme activity or *Sss*I-catalyzed cytosine-to-uracil conversion in SAM deficient condition (Bandaru et al., 1995; Zingg et al., 1996; Stier and Kiss, 2013). Nevertheless, the 30% methylation level acquired from the 1:1 mixed DNA template implied that the methylation frequencies of target CpGs at genomic regions were well-preserved even after the multiplex PCR and library preparation processes during TBPseq.

### Correlation Analysis of Targeted Bisulfite PCR Sequencing Data

**FIGURE 2:**
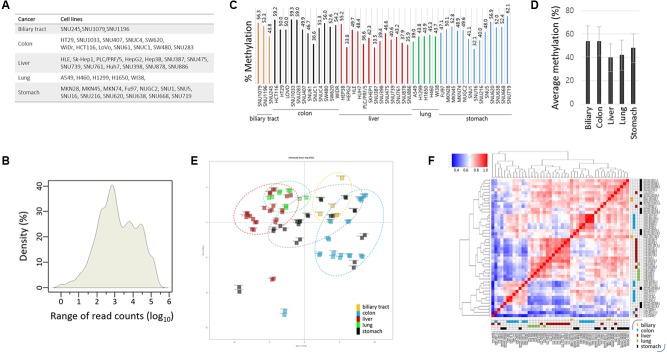
Targeted bisulfite PCR sequencing statistics and correlation analysis. **(A)** List of cancer cell lines used in TBPseq. **(B)** Kernel density plot of read counts from the whole cancer samples. Individual target sequences have various read counts ranging from several 10s to 100s of 1000s. The mean methylation levels of target sequences in individual cancer cell lines **(C)** and in groups **(D)**. Error bars, standard deviations. **(E)** Principal compartment analysis (PCA). Different cancer cell groups are circled in different colors. The intimate proximity of sample replicates on the PCA plot demonstrates the reproducibility and reliability of TBPseq method. **(F)** Unsupervised hierarchical clustering of Pearson correlation for target CpG methylation levels among cancer cell lines. Each cancer cell line is distinctively colored by the cancer type: yellow, biliary tract cancer; blue, colon cancer; red, liver cancer; green, lung cancer; black, stomach cancer.

The target CpGs were examined in 46 cancer cell lines—3 biliary tract, 12 colon, 13 liver, 5 lung, and 13 stomach cancer cell lines (**Figure 2A**). We randomly chose these five groups of cancer cell lines and selected cancer-related CpGs, unaware of their associations with certain cancer types. With these choices, we hoped that some of the previously established cancer-cDMC matches would be exposed by our TBPseq analysis. For each cell line, duplicated sequencing libraries was produced and all libraries, containing 92 barcodes in total, were all pooled for sequencing in a single flow cell. As a result, 1.6 × 10^8^ reads (1.6 × 10^6^ reads on an average per sample) in sum were obtained, with the average mapping efficiency of 79.7 ± 7.7%. Of the target amplicons, 78.8% had >100 read counts while 57.6% had >1,000. Overall, the mean coverage count was 9,256 (**Figure 2B**). Target amplicons with <100 read counts were removed from the methylation analysis because they were less reproducible between the replicates. Thus, methylation data analysis was performed for 165 CpGs from 67 gene promoters.

The mean methylation levels of the 165 target CpGs varied among the cell lines, ranging from 32 to 62% (**Figure 2C**). The group methylation levels are shown in **Figure 2D**; the colon cancer cell group showed the highest level (52.0 ± 6.7%) whereas the liver cancer cell group (40.7 ± 7.0%) showed the lowest level of methylation. Using the methylation levels of target CpGs, we assessed the degree of correlation among the cancer cell lines. Principal component analysis (**Figure 2E**) and unsupervised hierarchical clustering analysis (**Figure 2F**) showed that both the lung and liver cancer cell lines were gathered together and strongly correlated to each other, whereas the stomach cell lines were the most dispersed showing an intra-tumor heterogeneity. Correlations between the replicates were very high, as shown by their proximity on the PCA plot, which demonstrates the reproducibility and reliability of the TBPseq method. Pearson correlation values and scatter plots between the cancer cell line groups are shown in Supplementary Figure S2. The results showed that colon cancer cells were weakly correlated with liver and lung cancer cell lines (*r* < 0.77) while stomach cell lines were, interestingly, strongly correlated to all other cancer cell lines (*r* > 0.86), regardless of the highly heterogeneous feature among the within-group cell lines.

### Identification of Cancer-Specific Methylated CpG Sites

**FIGURE 3:**
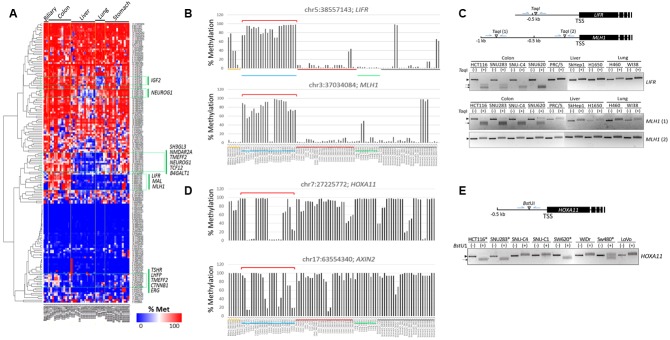
Identification of CpG sites specifically methylated in colon cancer cell lines. **(A)** Heat map of the methylation levels of target CpGs. Target CpGs that are differentially methylated among the cell lines are indicated by green boxes with the associated gene symbols. Methylation levels of the *LIFR* and *MLH1* **(B)** and *HOXA11* and *AXIN2* **(D)** CpG sites in colon cancer cell lines. Chromosomal locations of target CpG sites are represented as the chromosome (chr) number and coordinate of the position of cytosine. Cell lines of different cancer groups are indicated in different colors below: biliary tract, colon, liver, lung, and stomach cancer cell lines are yellow, blue, dark red, green, and black, respectively. Brackets (red) denote colon cancer cell lines showing differential methylation patterns. **(C,E)** Combined bisulfite restriction analysis (COBRA). Schematics show the relative positions of the transcription start sites (TSSs) of target genes and the restriction enzyme (*Taq*I or *Bst*UI) site. Genomic DNA was treated with bisulfite to convert unmethylated cytosines to uracils, which are then amplified as thymines, and then used as a template in PCR. The PCR products were digested with the indicated restriction enzyme. If the region of interest were methylated, the PCR product would be digested. In **(E)**, only colon cancer cell lines were included, and *HOXA11* promoter-methylated cell lines are indicated by asterisks. Arrow heads and arrows indicate the band positions of intact and enzyme-digested PCR products, respectively.

A heat map of the methylation levels revealed differentially methylated CpGs among the cancer cell lines (**Figure 3A**). Using DESeq in *R*, we identified cDMCs between the cancer cell groups, and we designated individual CpG sites by their genome position (GRCh37/hg19 as reference genome). In the comparison of the colon vs. the other cancer groups, chr5:38557143 (*FDR* = 9.25 × 10^-13^) and chr3:37034084 (*FDR* = 2.32 × 10^-11^) at the *LIFR* and *MLH1* gene promoters, respectively, were found to be differentially methylated; both CpGs were consistently hypermethylated in the colon cancer cell lines while hypomethylated in the other cell lines (**Figure 3B**). The *LIFR* CpGs were previously reported to be specifically methylated in colon cancer samples (Cho et al., 2011). The *MLH1* promoter was also shown to be heavily methylated in colon cancer samples (Kane et al., 1997; Cunningham et al., 1998; Herman et al., 1998). The colon cancer-specific methylation at *LIFR* and *MLH1* promoters was verified by combined bisulfite restriction analysis (COBRA), a method used to determine DNA methylation levels at a specific genomic locus using restriction endonucleases (Xiong and Laird, 1997; Kang et al., 2001, 2002). COBRA using *Taq*I (5′-TCGA-3′) showed that the *LIFR* promoter was methylated specifically in the colon cancer cell lines but not in the liver and lung cancer cell lines (**Figure 3C**). The distal region of the *MLH1* promoter, which corresponds to the 5′ shore of the CpG island and is adjacent to the identified *MLH1* cDMC (chr3:37034084), was shown methylated in a colon cancer-specific manner, whereas the proximal region residing within the CpG island was equally unmethylated in all cell lines examined.

Chr7:27225772 at the *HOXA11* promoter and chr17:63554340 at the *AXIN2* promoter were also detected as colon cancer-specific cDMCs. However, their methylation levels were not uniform, showing extremely polarized methylation states in the colon cancer cell lines (**Figure 3D**). For example, the *HOXA11* cDMC was either unmethylated (HT-29, Lovo, SNU61, SNU-C1, and SNU-C4) or heavily methylated (HCT116, SNU1033, SNU188, SNU407, and SW620) in the colon cancer cell lines. A similar polarization of methylation states among the colon cancer cell lines was observed at the *AXIN2* cDMC. COBRA experimental results confirmed a different methylation state at the *HOXA11* promoter among the colon cancer cell lines (**Figure 3E**).

**FIGURE 4:**
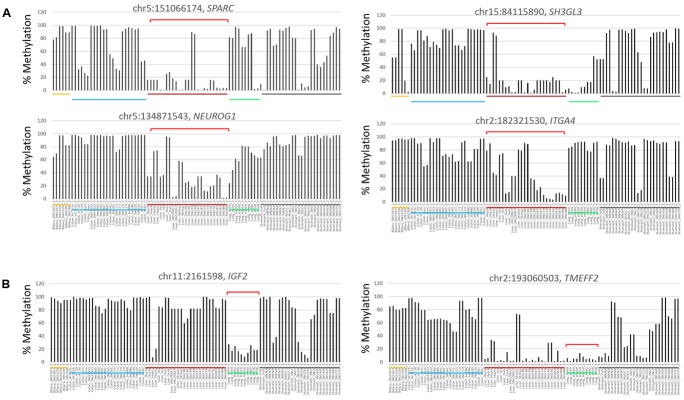
Differentially methylated CpG sites in liver and lung cancer cell lines. Target CpGs at the *SPARC, SH3GL3, NEUROG1*, and *ITGA4* gene promoters are frequently undermethylated in liver cancer cell lines **(A)** and so are those at the *IGF2* and *TMEFF2* gene promoters in lung cancer cell lines **(B)**. Brackets (red) denote those of cell lines showing group-specific methylation patterns for indicated CpGs. Chromosomal locations of target CpG sites are represented as the chromosome (chr) number and coordinate of the position of cytosine. Each cancer cell line group is underlined below the bars (yellow, biliary tract; blue, colon; red, liver; lung, green; black, stomach cells).

In the comparison between the liver cancer and the other cancer groups, cDMCs (*FDR* < 0.0001) were detected at the promoters of the *SPARC, NEUROG1, SH3GL3*, and *ITGA4* genes (**Figure 4A**). These cDMCs were frequently hypomethylated in the liver cancer cell lines. Lung cancer-specific cDMCs were found at the promoters of *SH3GL3*, *IGF2*, and *TMEFF2* (**Figure 4B**). No CpG site was identified to be specific for stomach cancer cells at *FDR* < 0.0001, which conformed to the heterogeneous character of stomach cancer cell lines (**Figures 2E,F**) and the higher correlation of the stomach cell lines with the other cancer cell lines (Supplementary Figure S2). **Table 1** lists the cancer-specific cDMCs and their associated genes.

**Table 1:** CpG sites differentially methylated among cancer cell groups.

Genes associated	CpG location^a^	Mean count	% Meth (± STD)^b^	FDR
**Colon cancer cell lines vs. others**				
*LIFR*	5:38557162	25837.86	82.10 ( ± 0.62)	2.21 × 10^-13^
	5:38557159			6.75 × 10^-13^
	5:38557143			9.25 × 10^-13^
*MLH1*	3:37033903	23971.48	69.50 ( ± 11.27)	3.19 × 10^-11^
	3:37033894			3.19 × 10^-11^
	3:37034084			2.32 × 10^-11^
	3:37034066			1.24 × 10^-6^
*HOXA11*	7:27225772	66302.95	52.07	1.61 × 10^-10^
*AXIN2*	17:63554340	81734.54	70.93 ( ± 0.61)	1.10 × 10^-8^
	17:63554353			1.67 × 10^-7^
*PPARG*	3:12329011	52496.36	40.96	1.19 × 10^-5^
**Liver cancer cell lines vs. others**				
*SH3GL3*	15:84115855	34259.59	29.03 ( ± 1.44)	1.30 × 10^-13^
	15:84115890			1.87 × 10^-9^
	15:84115895			1.45 × 10^-5^
*SPARC*	5:151066174	45657.91	23.92	8.03 × 10^-12^
*NEUROG1*	5:134871543	63209.05	34.05 ( ± 11.02)	2.28 × 10^-10^
	5:134871539			1.08 × 10^-7^
	5:134871515			1.67 × 10^-7^
	5:134871510			4.10 × 10^-7^
	5:134871516			1.23 × 10^-6^
	5:134871509			4.44 × 10^-6^
	5:134871544			2.67 × 10^-5^
	5:134871540			4.68 × 10^-5^
*RB1*	13:48877655	2194.00	11.73	2.45 × 10^-6^
*ITGA4*	2:182321530	61811.37	39.17	6.54 × 10^-6^
*NPM1*	5:170814340	2871.80	16.06 ( ± 3.79)	1.09 × 10^-5^
	5:170814379			7.65 × 10^-5^
*RB1*	13:48877641	2047.34	7.95	1.09 × 10^-5^
*NMDAR2A*	16:10276119	28688.10	13.47	1.43 × 10^-5^
*CDKN2A*	9:21975739	58680.32	77.24	3.93 × 10^-5^
*CBLB*	3:105587507	2925.30	19.60	3.96 × 10^-5^
*KLF5*	13:73632762	89833.42	81.36	5.18 × 10^-5^
*PAX3*	2:223163989	64484.74	43.06	6.30 × 10^-5^
**Lung cancer cell lines vs. others**				
*IGF2*	11:2161605	67594.01	26.45 ( ± 2.24)	4.66 × 10^-22^
	11:2161598			2.82 × 10^-20^
	11:2161586			5.51 × 10^-13^
*TMEFF2*	2:193060503	35910.80	5.60	2.42 × 10^-7^
*FHIT*	3:61237270	582.14	2.90	7.29 × 10^-6^
*LHFP*	13:40177664	15799.21	0.50 ( ± 0.08)	1.91 × 10^-5^
	13:40177654			9.83 × 10^-5^

*^a^Chromosome number and location is separated by colon. It refers to GRCh37/hg19 human reference genome. ^b^Mean % methylation ± standard deviation.*

### Meta-analysis of cDMCs and Functional Validation

**FIGURE 5:**
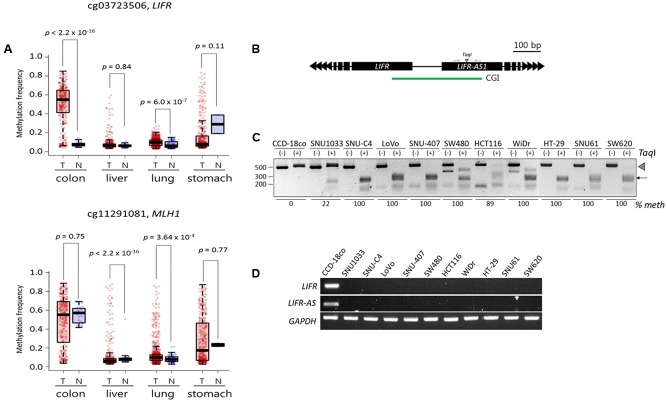
Validation of the *LIFR* promoter methylation for cancer specificity and its relationship with the expression of associated genes. **(A)** DNA methylation levels of target CpGs in public cancer methylome data (Infinium 450K BeadChip array) of four cancer types: colorectal (*n* = 313 for cancer samples and *n* = 38 for normal samples), liver (377 and 50), lung (843 and 74), and stomach (395 and 2) cancers. Infinium CpG identification numbers (IDs) together with the associated gene names are shown; the Infinium IDs cg03723506 and cg11291081 indicate chr5:38557143 and chr3:37033894, respectively, in the **Figure 3B**. Statistical significance was calculated using Wilcoxon rank sum test. T: tumor samples, N: normal samples. **(B)** Schematic drawing of COBRA region at the *LIFR* promoter. Blue arrows, primers. CGI, CpG island (green line). **(C)** COBRA analysis. Genomic DNA was extracted from each colon cancer cell lines along with a normal control colon cell line (CCD-18co) and subjected to COBRA using the *Taq*I enzyme to examine the methylation state at the *LIFR* gene promoter. Arrowhead and arrow indicate the positions of intact and *Taq*I-digested DNA fragments, respectively. The fraction (% meth) of methylated DNA was measured by band intensity analysis and noted under each cell line. **(D)** RT-PCR. The same cancer cell lines used in COBRA **(C)** were subjected to RT-PCR to measure the transcript levels of the *LIFR* and *LIFR-AS* genes.

We performed a meta-analysis of publicly available DNA methylome data from colon (*n* = 313 for cancer samples and *n* = 38 for normal samples), liver (377 and 50), lung (843 and 74), and stomach (395 and 2) cancer patient samples in Genomic Data Commons portal (Supplementary File 2). In line with our TBPseq results, the cg03723506 and cg11291081 CpGs, which represent the Infinium (Illumina) CpG identification numbers^3^ and are the same CpGs with the *LIFR* and *MLH1* cDMCs, respectively (see the **Figure 5** legend), showed a heavier methylation in a colon cancer-specific manner (**Figure 5A**). In the case of *LIFR* cg03723506, the difference in methylation level between the colon cancer and normal samples was remarkable (*p* < 2.2 × 10^-16^; Wilcoxon rank sum test). However, no significant differential methylation patterns at *MLH1* cg11291081 was observed between the normal controls (55.5%) and the colon cancer samples (48.9%; *p* = 0.75). We also found the expression of *MLH1* mRNA in the most colon cell lines whose cg11291081 were methylated (data not shown). Thus, the result indicates that the hypermethylation at *MLH1* cg11291081 in the colon cancer cell lines occurs in a tissue-specific fashion, rather than in a cancer-specific fashion.

We next examined if the *LIFR* cg03723506 methylation was linked to the expression of the associated genes. We analyzed the colon cancer cell lines (**Figure 2A**) along with a normal colon cell line (CCD-18co) for *LIFR* promoter methylation and mRNA expression. The *LIFR* cDMC is located at the promoter region shared by the *LIFR* and *LIFR*-AS genes (**Figure 5B**). The COBRA result showed that the *LIFR* cDMC was unmethylated in the CCD-18co control cells whereas it was heavily methylated in most colon cancer cell lines (**Figure 5C**). RT-PCR result showed that both the *LIFR* and *LIFR-AS* transcripts were detected in the normal CCD-18co cells only but not in the other colon cancer cell lines (**Figure 5D**). Other non-colon cancer cell lines such as SNU449 liver cells which were not methylated at the *LIFR* cg03723506 cDMC also expressed the *LIFR* and *LIFR-AS* genes (Supplementary Figure S3). This result suggests an intimate negative correlation between the *LIFR* cg03723506 hypermethylation and the expression of its associated genes.

## Discussion

We developed a PCR-based targeted sequencing approach that is optimized for DNA methylation analysis. We targeted 246 CpG sites in this study, but the size and scale could be expanded, if necessary. The panel composition is highly flexible and can accommodate a variety of experimental designs, a big advantage over other methylation analysis platforms such as the array-based Infinium BeadChip. The WGBS method is, in general, conducted with a limited number of samples with coverage usually ranging from 5 to 15 times per CpG, which limits the statistical significance of the findings. Compared to this WGBS, our TBPseq method can accommodate large numbers (several 10s or more) of samples in the same flow cell and produce 10s of 1000s of read counts on an average per target CpG. In addition, TBPseq uses only selected targets for analysis, instead of the whole genome as a target, which saves time during the data analysis. As a TBPseq-like targeted methylation analysis approach, a bisulfite padlock probes sequencing (BSPP-seq) method is featured by targeted capture of bisulfite-converted DNAs, the flexibility in selecting targets, and its library-free sequencing protocol (Diep et al., 2012). However, the engine for designing padlock probes and the matching bioinformatics pipeline are unfamiliar, and the requirement of a large amount (>200 ng) of sample DNA in single BSPP-seq may limit its application to certain clinical diagnosis. Taken together, our TBPseq represents a cost-effective, high-throughput, and time-saving method capable of single-base resolution analysis. With these benefits, the TBPseq will, we hope, facilitate the exploration and development of methylation biomarkers and conduce to the improvement of cancer detection in clinics.

Targeted bisulfite-PCR-sequencing produced a broad range (from several 10s to 150,000) of reads per target, which is likely to depend on the annealing strengths of primer pairs. Primer pairs with a low performance pose a problem, leading to insufficient reads; however, those with an extremely high performance could also be problematic because vigorous amplifications of target sequences by strong primers could indirectly interfere with the weak primer-mediated amplifications of other target sequences by exploiting most of the PCR resources (McPherson and Hames, 1995). This is an especially serious concern when performing multiplexed PCR (Sninsky et al., 1999). We are currently trying to devise a method to evenly produce target amplicons, for example, by optimizing combinations of primer pairs for multiplexing. Fortunately, this is feasible because our TBPseq primer panel is highly flexible and easily reconfigurable to various situations and requests.

We designed multiplexing primers following the principle that each amplicon should be 60–100 bp in length and contain a single CpG site, if possible. This is because the presence of multiple CpG sites in a small-sized target sequence could prevent the accurate assessment of methylation frequency, as the base composition and the GC content of template DNA after bisulfite conversion would greatly differ by the methylation level. However, the requirement of minimal CpGs within the amplicon sequences for TBPseq analysis raises questions regarding which CpG should be preferred among many adjacent CpGs and how representative the selected CpG(s) would be for the region of interest. This is an important concern especially when a methylation analysis platform is used to examine a group of representative CpGs, as in TBPseq and Infinium BeadChip. There were, in fact, some infrequent cases in which two adjacent CpGs within a single amplicon were oppositely methylated (unpublished observation). A possible explanation of this is that a protein may bind to the DNA region to block methylation one of the two CpGs. Therefore, although such cases were scarce and restricted to only certain regions, based on our results, inclusion of multiple CpGs as targets are recommended for the accurate measurement of the methylation level of a certain genomic region as well as adherence to rigorous validation processes. In order to choose an appropriate CpG, the one that has an Infinium BeadChip CpG ID number would be a better choice, because the majority of public methylome data have been obtained from the Infinium BeadChip platform and are referred to by their Infinium CpG ID. By sharing the same CpGs with the Infinium platform in the methylation analysis, our data could be directly and conveniently compared with the big public data.

In this study, we separately and randomly selected five groups of cancer cell lines and 246 cancer-related CpGs, irrespective of the specific relationships of certain CpGs to certain cancer types, among them. Because of this, we could perform a blind experiment to test whether TBPseq is able to expose some known cDMC-cancer type associations that are well-established as cancer methylation markers, the results of which could demonstrate the competence of the TBPseq method. Conforming to our expectation, TBPseq succeeded in determining the colon cancer-*LIFR* CpG matches, which demonstrates high efficiency of TBPseq technology in target-based high-throughput methylation analysis.

As shown in **Figure 3**, the *LIFR* promoter methylation was detected only in the colon cancer cell lines and not in other cancer cell lines such as biliary tract, liver, lung, and stomach cancer cells. This result is supported by a previous report of colon cancer-specific methylation at the *LIFR* promoter (Cho et al., 2011), although that study was not expanded to survey other cancer samples. Our results from the analysis of cancer cell lines (**Figures 5C,D**) as well as the meta-analysis of public DNA methylome data (**Figure 5A**) unambiguously showed increased methylation levels at the *LIFR* gene promoter in colorn cancer samples compared to other types of cancers and normal colon samples. Furthermore, the promoter methylation correlated with downregulation of the *LIFR* gene expression, which leads to a speculation of the importance of *LIFR* suppression in the development of colon cancer development. Our results suggest that the *LIFR* cDMCs may not only be used as a DNA methylation biomarker for cancer identification, but by extension, for cancer typing as well.

In addition to the *LIFR* cDMC, the *MLH1* cg11291081 cDMC was found heavily methylated in the colon cancer cell lines while it was only barely methylated in the other cancer cell lines (**Figure 3C**), which misled us to consider it as a colon cancer-specific methylation marker. We finally concluded the cg11291081 was variously methylated in a tissue-specific manner after we found it was ∼50% methylated in the normal colon samples but ∼8% in the normal liver and lung samples (**Figure 5A**). Although the *MLH1* cg11291081 hypermethylation turned out to be tissue-specific, instead of cancer-specific, it did not mean that our TBPseq failed its task, because the method succeeded in pointing the cg11291081 anyway as a differential marker specific to the colon cancer cell lines against the other cancer cell lines. Meanwhile, the hypermethylation at the *MLH1* promoter has been well-known in colon cancers (Kane et al., 1997; Cunningham et al., 1998; Herman et al., 1998; Simpkins et al., 1999; Toyota et al., 1999; Nakagawa et al., 2001; Wallner et al., 2006; Weisenberger et al., 2006; Ahlquist et al., 2008; Hinoue et al., 2012; Li et al., 2013). These earlier studies primarily analyzed the body of *MLH1* promoter CGI, whereas the cg11291081 site examined in this study resides in the shore of the CGI. The different CpG positions may explain the different results between ours and those earlier studies.

In the very near future, through meta-analysis and co-methylation analysis of public cancer methylome data, packs of cDMC markers that are significantly associated with certain types of cancers could be collected. We envision targeting these groups of cDMC markers and using TBPseq to analyze patient cancer samples. Those results could serve as important reference material for determining cancer diagnosis and prognosis, and could be extended for the development of a therapeutic plan.

## Author Contributions

KJ and JP performed the experiments. BM and KJ carried out bioinformatic and statistical analysis. Y-KK and KJ had designed the experiments, interpreted the results and written the manuscript. All authors read and approved the final manuscript.

## Conflict of Interest Statement

The authors declare that the research was conducted in the absence of any commercial or financial relationships that could be construed as a potential conflict of interest.

## Footnotes

1 https://www.r-project.org/

2 https://portal.gdc.cancer.gov

3 https://support.illumina.com
