# Supplementary material for: Simultaneous Methylation-Level Assessment of Hundreds of CpG Sites by Targeted Bisulfite PCR Sequencing (TBPseq)
Source: Front Genet. 2017 Jul 13;8:97. doi: 10.3389/fgene.2017.00097 (PMC5507944; doi:10.3389/fgene.2017.00097)
Supplement: Supplementary file 3 [file Image_1.PDF]

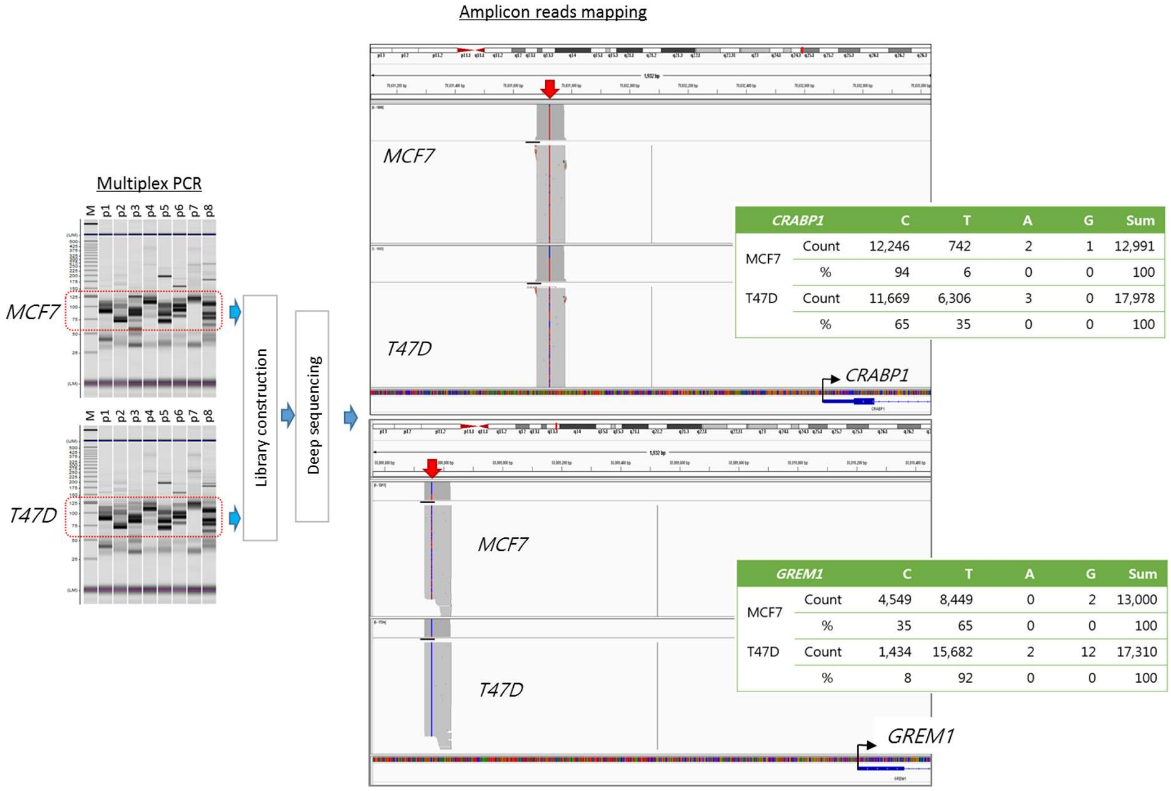

Supplementary Figure S1. Schematic procedure of the preliminary tests of TBPseq using MCF7 and T47D, breast cancer cell lines, and bioinformatics analyses for the quantification of DNA methylation level. Using bisulfite treated gDNAs of MCF7 or T47D cell lines, multiplex PCRs were performed, and the resulting amplicons were analyzed by MultiNA, a microchip electrophoresis system. Illumina NGS libraries were generated using the amplicons, and the sequenced reads were mapped on the human genome. The IGV images demonstrate the determination process of methylation level. Base compositions at target CpG sites were measured, and methylation levels were calculated by dividing the number of C's by the sum of C's and T's.
