# Supplementary material for: Simultaneous Methylation-Level Assessment of Hundreds of CpG Sites by Targeted Bisulfite PCR Sequencing (TBPseq)
Source: Front Genet. 2017 Jul 13;8:97. doi: 10.3389/fgene.2017.00097 (PMC5507944; doi:10.3389/fgene.2017.00097)
Supplement: Supplementary file 4 [file Image_2.PDF]

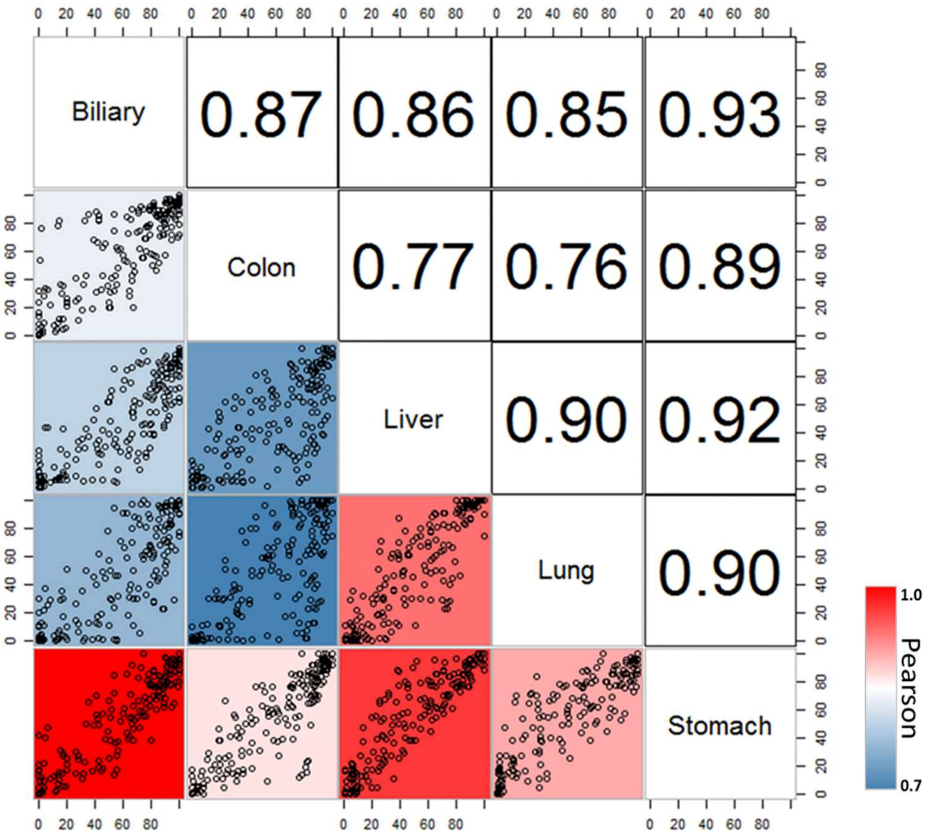

Supplementary Figure S2. Scatter matrix of Pearson correlation (*r*) analysis between different cancer types.
