# Supplementary material for: Simultaneous Methylation-Level Assessment of Hundreds of CpG Sites by Targeted Bisulfite PCR Sequencing (TBPseq)
Source: Front Genet. 2017 Jul 13;8:97. doi: 10.3389/fgene.2017.00097 (PMC5507944; doi:10.3389/fgene.2017.00097)
Supplement: Supplementary file 5 [file Image_3.PDF]

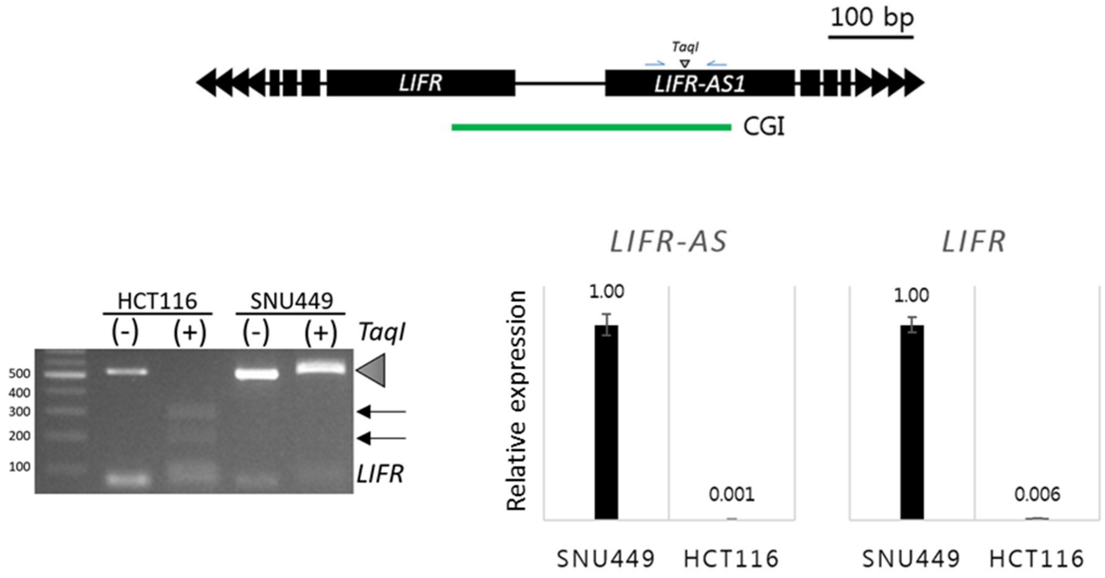

Supplementary Figure S3. Relationship of the *LIFR* promoter methylation with the expression of the associated genes. Left, COBRA analysis using genomic DNAs extracted from SNU449 liver and HCT116 colon cancer cell lines. Right, result of quantitative real-time PCR with the cDNA obtained from the same cells to measure the *LIFR* and *LIFR-AS* gene transcript levels.
